# Editorial: Neuromuscular adaptations to sensorimotor stimulation protocols: potential rehabilitative interventions for individuals with central or peripheral neuromuscular injuries

**DOI:** 10.3389/fresc.2024.1388989

**Published:** 2024-03-06

**Authors:** Jongsang Son, M. Hongchul Sohn, Christopher K. Thompson

**Affiliations:** ^1^ Department of Biomedical Engineering, New Jersey Institute of Technology, Newark, NJ, United States; ^2^ Department of Physical Therapy and Human Movement Sciences, Northwestern University, Chicago, IL, United States; ^3^ Department of Health and Rehabilitation Sciences, Temple University, Philadelphia, PA, United States

**Keywords:** rehabilitation, intervention, noninvasive brain stimulation, neuromuscular stimulation, stroke, spinal cord injury, neuromodulation, personalized rehabilitation

**Editorial on the Research Topic** Neuromuscular adaptations to sensorimotor stimulation protocols: potential rehabilitative interventions for individuals with central or peripheral neuromuscular injuries

Central or peripheral neuromuscular injuries may result in a range of impairments in motor function that can compromise an individual's ability to complete daily living tasks. While retraining voluntary tasks—whether through practice or using compensatory strategies—has been traditionally considered the main goal in routine clinics, there is an impending need for more effective rehabilitation strategies to improve clinical and functional outcomes. Recent evidence suggests that combined sensorimotor stimulation of various forms may lead to better functional outcomes (1–3). However, we currently lack a comprehensive understanding of how the neuromuscular system can be reshaped in response to various sensorimotor stimulations to improve overall motor function. Rather, studies up to date have largely focused on understanding neural pathway reorganization (4–7), altered muscle activation patterns (8–10), or muscle structural/architectural changes (11–13) following central or peripheral neuromuscular injuries in isolation. Understanding the neuromuscular adaptation to sensorimotor stimulation is required to develop more effective rehabilitation intervention protocols that can help individuals after central or peripheral neuromuscular injuries maximize functional outcomes.

The goal of this research topic is to compile a collection of scholarly articles that advance our current knowledge of the short- or long-term effects of sensorimotor stimulation protocols on the neuromuscular system in both neurologically intact individuals and those with central or peripheral neuromuscular injuries. In response to the call for contributions, five research articles were included in this collection; as means of probing into neuromuscular adaptation to sensorimotor stimulation, Thompson et al. employed operant conditioning of H-reflex, Eginyan et al. electrical stimulations, Palimeris et al. 4-week tailoring strengthening exercise, Sivertsen et al. 12-week physical therapy, and Cohn and Valero-Cuevas a computational simulation. Out of four experimental research, three articles reported their effects in acute (Sivertsen et al.) and chronic (Thompson et al. and Palimeris et al.) stroke survivors, and one in neurologically intact individuals (Eginyan et al.). We have summarized each article published in this Research Topic below.

Thompson et al. tested whether spinal reflex behaviors can be adapted through operant conditioning in individuals with stroke, i.e., supraspinal neural injuries. Specifically, the authors examined whether the application of operant down-conditioning protocol over 12 weeks (6 baseline and 30 conditioning sessions) can decrease the soleus H-reflex in individuals with cortical or subcortical stroke. Results varied among participants with stroke: H-reflex became significantly smaller in 6 of 12 participants but not in the other 6 participants. Functional outcome, as measured by an increase in 10-m walking speed also varied among the 6 participants in which H-reflex decreased whereas there was no change in the other half. Relatively low conditioning success rate (i.e., 50%) as compared to those previously found in individuals with incomplete spinal cord injury suggested that supraspinal activity may play an important role in inducing long-term plasticity in the spinal cord.

Eginyan et al. evaluated the effects of tibial nerve stimulation (TibNS) on corticospinal excitability of both the abductor hallucis (AH) muscle, directly innervated by the tibial nerve, and the pelvic floor muscle (PFM), sharing spinal segmental innervation with the tibial nerve, in healthy individuals. The authors also compared the effects of applying TibNS using continuous (likely employed in clinical settings) vs. intermittent (likely employed in research settings) patterns on corticospinal excitability of these muscles. The results demonstrated that regardless of stimulation patterns, TibNS significantly increased corticospinal excitability of AH (i.e., target muscle), but no effect was observed on corticospinal excitability of PFM (i.e., non-target muscle).

Palimeris et al. investigated the effects of 4-week tailoring strengthening exercises based on corticospinal integrity in chronic stroke survivors by conducting a multisite randomized controlled trial. The authors also tested the effects of applying anodal transcranial direct current stimulation (tDCS) in addition to the tailoring strengthening exercise. The results demonstrated that regardless of training intensity, primary outcomes including the Fugl-Meyer assessment, box and block test, and grip strength were significantly improved after the tailored training intervention. However, the tailored training intervention in combination with tDCS had no significant impact on outcomes post-intervention.

Sivertsen et al. demonstrated the results of a randomized controlled trial that compared the effects of a 12-week comprehensive physical therapy intervention, called I-CoreDIST, with usual care physical therapy on various outcomes in acute stroke survivors. I-CoreDIST incorporates individualized exercises that specifically aim to improve balance, gait, upper limb function, and functional activities. Both I-CoreDIST and usual care physical therapy led to significant improvements in postural control, balance, physical activity, and gait during the first 12 weeks after stroke, showing no significant differences between the two therapy groups.

Cohn and Valero-Cuevas employed computational simulations to probe into how inherent temporal constraints imposed by muscle activation-contraction dynamics shape the feasible motor solution space in the fingertip force production task. Without temporal constraint, that is, assuming a static linear mapping between muscle activation and output endpoint wrench (force and moment), the space of all possible motor commands to achieve the same task was highly redundant. However, as temporal limits on muscle activation-contraction dynamics were added, the redundancy was substantially reduced, only allowing for a narrower choice of motor commands in sequence available. Based on their results, the authors discuss a theoretical framework for how changes in the rate of muscle force production can structure the landscape of feasible motor commands for a given task after central or peripheral neuromuscular injuries and training.

This collection supports a well-expected perspective that neuromuscular adaptation to sensorimotor stimulation is multi-faceted. It is important to recognize that individual responses to the proposed intervention protocols vary, with some being responders and others non-responders. For example, a significant reduction in the H-reflex amplitude was observed in six of the twelve chronic stroke survivors (Thompson et al.), and functional improvement was found in 70 of the 90 chronic stroke survivors (Palimeris et al.). While the authors discussed the potential factors (e.g., alterations in the supraspinal pathway and baseline characteristics) in part, the contribution of other factors, including genetics and lifestyle, remains largely unexplored. This underscores the need for implementing precision or personalized rehabilitation strategies. In this regard, two studies in this collection (Palimeris et al. and Sivertsen et al.) utilized the participants' pre-evaluation data to tailor the intervention protocols, but these modifications did not result in a significant difference in the primary outcomes. Given that individuals' level of function needs to be assessed via precise measurement of their abilities across multiple domains (e.g., physical, cognitive, and psychosocial factors) (14), future studies may adopt a more comprehensive approach that can lead to collecting additional, multidisciplinary outcome measures, evaluating the effects of intervention protocols on each individual and thus elucidating potential associations of individual responses, encompassing both element-wise (e.g., neural, sensory, and musculoskeletal responses) interaction and system-level changes.

To this end, a range of methodologies must be employed to investigate this individualized system. Even within the five manuscripts in this Research Topic, there is a range of approaches including nerve electrical stimulation, transcranial magnetic stimulation, physical therapy, and computational simulation as means of investigating neuromuscular adaptation to sensorimotor stimulation. We hope that these collective efforts will contribute to advancing the field of rehabilitation science toward developing more effective interventions as well as personalized rehabilitation.
